# Diagnostic Dilemmas of Wilson's Disease

**DOI:** 10.7759/cureus.79612

**Published:** 2025-02-25

**Authors:** Harsha Vardhini, Ganapathiearaman Ramakrishnan, Bhuvana Krishna

**Affiliations:** 1 Critical Care Medicine, St. John's Medical College and Hospital, Bengaluru, IND

**Keywords:** acute liver failure (alf), coomb's negative, haemolytic anaemia, leipzig score, serum ceruloplasmin, wilson`s disease

## Abstract

Wilson's disease is a rare autosomal recessive disorder characterized by impaired hepatic copper transport, leading to copper accumulation in tissues and diverse clinical manifestations. A 27-year-old male chemical factory worker presented with acute haemolytic anaemia, pancytopenia, jaundice, and altered sensorium, indicative of acute liver failure. Despite normal serum ceruloplasmin levels, elevated 24-hour urinary copper levels (227.53 µg/24 hours) supported the diagnosis of probable Wilson’s disease. The patient responded to symptomatic management, including transfusions and antibiotics, with gradual clinical and biochemical improvement before discharge against medical advice. This case underscores the diagnostic challenges of Wilson’s disease due to nonspecific symptoms and limitations of laboratory tests. It highlights the importance of clinical suspicion and a multidisciplinary approach to ensure accurate diagnosis and timely treatment.

## Introduction

Wilson's disease is a rare autosomal recessive disorder characterized by impaired hepatic copper transport. There is copper accumulation in tissues leading to acute liver dysfunction and central nervous system manifestations [1,2]. The prevalence of Wilson’s disease is 30 per million population which has progressively increased over the recent years [3,4,5,6]. Ceruloplasmin, a copper-binding protein synthesized in the liver, is typically decreased in Wilson's disease and serves as one of the diagnostic markers. There are a lot of challenges associated with a diagnosis of Wilson’s disease, mostly due to nonspecific clinical presentation and limitations of laboratory parameters. A patient presented with complex diagnostic challenges and was ultimately diagnosed with probable Wilson's disease. We present a case of a 27-year-old male, who was a worker in a chemical factory and had a history of exposure to unknown chemicals. His initial presentation to the hospital was suggestive of acute haemolytic anaemia and pancytopenia. The workup was not suggestive of haematological cause for haemolytic anaemia , and the evaluation for acute liver failure showed normal ceruloplasmin, with elevated urinary copper levels. The clinical and available lab parameters were suggestive of possible Wilson’s disease.

## Case presentation

A 27-year-old male came with complaints, of facial puffiness, yellowish discoloration of skin, and fatigue for four days. He was evaluated in an outside hospital, where he was found to have severe anaemia, pancytopenia, and high lactate dehydrogenase (LDH). Since the patient had severe anaemia, thrombocytopenia, altered liver enzymes, and persistent fever, he was referred to a higher center for further workup and treatment. On arrival at our hospital, the patient was in an altered sensorium and was hypoxic. He was intubated, initiated on mechanical ventilation in view of poor sensorium with a GCS of 7, stabilized, and shifted to the intensive care unit (ICU). 

In the ICU, the patient was continued on mechanical ventilation, hemodynamic supports, and routine ICU care. On examination, the patient was moderately built and well nourished, with icterus pallor, and no oedema or lymphadenopathy. His initial blood works were suggestive of severe pancytopenia with reticulocytosis (Table 1).

**Table 1:** Haematological profile. Hb: Haemoglobin, reference range (13.5–17.5 grams per deciliter (g/dL)); INR: international normalized ratio; mcL: platelets per microliter, reference range (150,000 and 450,000 platelets per microliter).

Days	Hb (g/dl)	Platelets (mcL)	INR
D1	2.8	14,000	2.27
D2	2.7	25,000	1.99
D3	5.7	20,000	1.15
D4	8.7	27,000	1.99

His liver function test revealed severe liver dysfunction, hyperbilirubinemia, transaminitis, elevated LDH, and coagulopathy (Table 2). Acute onset altered sensorium, and coagulopathy with jaundice were suggestive of acute liver failure. He was initially managed with multiple transfusions with packed red blood cells for correction of anaemia and coagulopathy. 

**Table 2:** Liver function tests and lactate dehydrogenase. TB: total bilirubin, reference range-0.1 and 1.2 milligrams per deciliter (mg/dL); DB: direct bilirubin, reference range-less than 0.3 milligrams per deciliter (mg/dL); IB: indirect bilirubin, reference range-less than 0.3 milligrams per deciliter (mg/dL); AST: aspartate aminotransferase, reference range-8 to 33 units per liter (U/L); ALT: alanine aminotransferase, reference range-4 and 36 units per liter (U/L); ALP: alkaline phosphatase, reference range-44 and 147 international units per liter (U/L). mg/dL: milligrams per deciliter; U/L: units per litre; LDH: lactate dehydrogenase, reference range-44 and 147 international units per liter (U/L).

Days	TB	DB	IB	AST (U/L)	ALT (U/L)	ALP (U/L)	LDH (U/L)
D1	8.42	1.42	7	3170	1976	73	6422
D2	16.61	4.5	12.6	1866	1773	70	3456
D3	18.4	8.0	8.01	504	979	70	2365
D5	11.68	7.5	7.57	28	234	78	2365

The patient was worked up for causes of acute haemolysis and acute onset liver failure including tropical infections. His direct Coombs test was negative, and his anaemia workup remained inconclusive. He had a reticulocyte count of 0.51 which improved to 1.18 by day 5. Leukopenia and thrombocytopenia were seen in the peripheral smear. He underwent bone marrow aspiration and biopsy which was reported as hypercellular marrow with erythroid hyperplasia and megaloblastic picture. Haematologist opinion was sought and advice was followed.

During the course in the ICU, in view of persistent fever, and elevated procalcitonin he was initiated on broad-spectrum antibiotics, which was titrated as per the culture reports. As a part of the haemolytic work up patient’s serum ceruloplasmin levels and 24-hour urinary copper levels were sent. His serum ceruloplasmin levels were normal.

The patient started to respond to symptomatic management. His sensorium improved, and with stable heamodynamics, and resolution of fever, he was weaned off from the ventilator and extubated. He later was shifted under the care of Haematology. He gradually showed improvement in liver parameters and blood parameters. Due to unavoidable reasons, the family discharged the patient against medical advice.

 On further follow-up of this patient’s records, the urinary copper analysis was reported to be high, (227.53 microgram per 24 hours) which was as high as two times the upper limit of urine copper levels (normal: 15-60 µg/24 hours). Therefore, a patient with acute onset non-haemolytic anaemia and acute liver failure was diagnosed to have possibly Wilson’s disease even though he had normal serum ceruloplasmin levels, based on clinical presentation and elevated urinary copper excretion as per the currently available diagnostic scoring system.

## Discussion

Wilson’s disease is an inherited disease with defective biliary excretion of copper, leading to copper deposition in the liver and brain [1,2]. Key features are cirrhosis of the liver associated with neuropsychiatric manifestations and Kayser-Fleischer (KF) ring in the eyes. The clinical features like KF rings may be present in 95% of patients having neurological symptoms but may also be seen in patients with cholestatic jaundice due to other causes [7,8]. The liver involvement of Wilson’s disease may vary from asymptomatic enzyme elevation to cirrhosis. Sometimes they may present with hyperacute liver failure which warrants early diagnosis and liver transplantation [9]. They may also have acute haemolysis, typically Coombs negative haemolysis. This can be acute episodic haemolysis, or recurrent low-grade chronic haemolysis [10].

Patients can present with only haemolytic symptoms, many times causing acute liver dysfunction. The neurological spectrum of the disease ranges from extrapyramidal symptoms in the form of dysarthria, dystonia, gait abnormalities, ataxia, tremor, parkinsonism, and chorea. Pyramidal and cerebellar involvement may also be seen. The behavioural changes and psychiatric symptoms can be in the form of mood disorders (depressive or bipolar spectrum), psychotic disorders, sleep disturbances, and subtle cognitive dysfunction [11]. Patients presenting with neuropsychiatric symptoms, and cirrhosis with KF ring in eyes are classical descriptions of Wilson’s disease. However, this presentation occurs in only 50% of cases [7]. According to current recommendations, any patient presenting with acute liver failure and Coombs-negative hemolytic anemia should be evaluated for Wilson’s disease. Our patient exhibited Coombs-negative hemolytic anemia with acute liver failure, where liver failure was secondary to acute hemolysis [11].

The lab workup of patients with Wilson's disease is accompanied by many diagnostic challenges. The liver function test may show only subtle elevation in liver enzymes (AST/ALT), disproportionate to the severity of liver involvement. Ceruloplasmin is a 132k-Da glycoprotein synthesized in the hepatocytes. Copper containing ceruloplasmin accounts for 90% of circulating copper. It is measured using enzymatic activity or immune assays. It is a non-specific test for screening or diagnosing Wilson's disease due to its limitations. The immune assays tend to overestimate ceruloplasmin levels. Ceruloplasmin is an acute phase reactant that can be elevated in the background of acute inflammation. Typically ceruloplasmin levels are reduced in Wilson disease. The other conditions with low ceruloplasmin levels are zinc excess, protein-losing enteropathy, Menke’s disease, and post-gastric bypass or bariatric surgeries [12,13,14]. However normal ceruloplasmin level does not rule out Wilson’s disease. The cut-off used for ceruloplasmin levels may also vary and influence the results. A 24-hour urinary copper excretion reflects the amount of non-ceruloplasmin-bound copper in circulation. Spot urine specimens are not reliable, except in acute liver failure where high urinary excretions might be expected. Other liver conditions like autoimmune haemolytic disease, and primary sclerosing cholangitis are also associated with excess urinary copper, therefore results cannot be reliable when used alone [11].

The other tests available are serum uric acid, serum copper levels, non-ceruloplasmin-bound copper, and radiocopper studies. Liver biopsy and hepatic parenchymal copper concentration are invasive methods available for confirmation of diagnosis of Wilson’s disease. Genetic tests for Wilson’s disease can be done for screening a first-degree relative or as a confirmatory test in suspected cases. CT/MRI can be used to screen for copper deposition in CNS. All these tests have a limitation of non-specificity and can’t be used as sole evidence to rule out Wilson’s disease [11].

A scoring system was developed considering the clinical presentation and biochemical tests. Later it was strengthened by adding genetic testing. This has been validated in both adult and paediatric populations. As per the scoring system, our patient had a score of 3, which makes Wilson’s disease a possibility and warrants further test for confirmation [11].

## Conclusions

Wilson's disease can present clinically as liver disease, a progressive neurological disorder, a psychiatric illness, or a combination of all these. It carries a mortality burden of almost 80-99%, requiring early liver transplantation. The presence of acute liver failure, with haemolytic anaemia, and neurological symptoms in a young adult should raise suspicion of Wilson's disease. Not a single biochemical test can rule in or rule out Wilson's disease. Therefore, diagnosing Wilson’s disease requires integrating clinical, biochemical, and genetic factors to accurately piece together the diagnostic puzzle.
